# When Lights Can Breathe: Investigating the Influences of Breathing Lights on Users’ Emotion

**DOI:** 10.3390/ijerph192013205

**Published:** 2022-10-13

**Authors:** Junfeng Wang, Jingjing Lu, Zhiyu Xu, Xi Wang

**Affiliations:** School of Design and Innovation, Shenzhen Technology University, Shenzhen 518118, China

**Keywords:** lights emotions, breathing light, emotion, physical indications

## Abstract

Light can significantly influence users’ physiological and behavioural performance. However, how light breathing influences users’ mood regulation remains unknown. To fill this gap, this study conducted a 2-by-2 experiment (N = 20) with light breathing as the between-subject factor and light condition as the within-subject factor. Both physiological indicators and subjective indicators are measured to reflect mood regulation. The data were analysed using a generalised linear mixed model. The results showed that breathing lights are effective in regulating users’ moods. More specifically, breathing lights help users lower their electrodermal values and heart rates. Users did not report any significant difference in terms of subjective measures, which suggest that the influence of a breathing light happens unconsciously. Furthermore, this effect is significant for both cold and warm colour temperatures. Designers and engineers can use the research findings to manage user emotion when necessary.

## 1. Introduction

Light has a direct impact on human physiological performance. The influences of light intensity, spectrum, and colour temperature have been extensively investigated in the current literature [1,2,3,4,5]. The research findings have been widely applied in a variety of fields, including medicine, transportation, and marketing communication [6,7,8]. Hartstein et al. found that adolescents exposed to low correlated colour temperature (CCT) had better sleep quality and were less exhausted the next day [9]. A study by van Bommel and van den Beld showed that switching from 300 lux to 500 lux illumination increased productivity in the metal-working industry by approximately 8%, whereas switching from 300 to 2000 lux generated a 20% improvement in productivity [10]. Dynamic light environments, which change in response to the natural environment and the physiological state of the human body, are a hot topic of research and have been shown to improve road safety [7].

Thus, how lighting influences users’ physiological and emotional responses becomes a crucial issue.

### 1.1. Light Environment

Light can not only change physical environment but also influence a person’s biological indicators such as circadian rhythms, body temperature, heart rate, cortisol, melatonin, etc. [10,11] Current studies have shown that the third photoreceptor cell (ipRGC) is able to connect directly to two parts of the brain: the suprachiasmatic nucleus (SCN) and the pineal gland [12]. The pineal gland is the gland in charge of hormone regulation in the body [13].

More specifically, because photoreceptor cells are sensitive to different wavelengths of light, people are sensitive to different colours of light. The wavelength band 446–477 nm is thought to be the most efficient in terms of delivering circadian input for the control of melatonin secretion [1]. Long wavelength light can boost alertness without interfering with melatonin secretion [2]. Blue light could help those performing multiple task representations to remain active and make less mistakes [14]. In turn, red light can be used to reduce daytime sleep inertia and improve short-term performance [15,16]. In addition, some findings highlighted the role of CCT in regulating instant emotion perception and showed lower CCT significantly decreased negative response bias in the face judgment tasks [17].

Furthermore, it has been demonstrated that light intensity has a significant effect on organisms’ night-time temperature and melatonin secretion [18,19]. Light’s ability to inhibit melatonin depends, to some extent, on light intensity and duration [20,21]. Subjects were exposed to bright light for 3 days, which could result in increased melatonin and decreased rapid eye movement (REM) sleep time [22]. This also implies that people who are exposed to bright light are more alert and perform better at night than those who are exposed to dim light [3]. Moreover, those who were exposed to bright light (approximately 2500 lx) before sleep had a significantly higher core body temperature while sleeping than those who were exposed to dark light (approximately 6 lx) [23]. This effect is not affected by the sleep cycle [24].

With these existing findings, light environment has been studied as a specific part of leading the emotions of crowds. For example, the luminaires used for exhibition—with different types, sources, sizes, beam angles, and CCT—could affect how participants feel about the artworks [25].

Despite extensive studies recognizing the significant influence of light on emotion, how light intervenes in users’ intense emotion remain unknown. Early research suggests that light appears to be useful in managing common mood and insomnia disorders [26,27]. This may be related to the phase advance of the melatonin rhythm [28,29]. Some studies have found that bright light therapy had a substantial therapeutic effect on depression in dementia patients, regardless of depression severity [30]. Dawn simulation is thought to result in higher rates of remission and response for seasonal affective disorder (SAD) compared with bright light therapy [31]. In summary, using light to alleviate negative emotions is promising. This study aims to investigate how light breathing influences users’ emotional responses.

### 1.2. Breathing

Rhythm is normally perceived by the human body throughout periodic regular events [32]. Rhythm is often used in auditory tasks. In Broadbent et al.’s study, auditory concurrent tasks interfered with children’s learning, while increased visual information helped focus children’s attention [33]. Voice interaction is thought to improve user perception and trust [34], although prior research suggested visual stimuli outperform auditory stimuli and can provide a distinct advantage in terms of behavioural dominance [35]. However, research on visual rhythm perception is still rare. Early studies explored visual rhythm through the use of flashes of light [36]. It was also discovered that flashes were effective in reducing the pain of intravenous injections and that this effect was significantly correlated with light colour [37,38]. Nevertheless, it has been demonstrated that humans perceive visual modal rhythms poorly in comparison with auditory rhythms, which may be due to the short visual perceptual memory time (0.3–1.0 s) [32].

Compared with the flashing light, the presentation effect of a breathing light is continuous and slow. It creates rhythmic pulsating light dimming and illuminating in synchronization with human breathing. Ståhl, A. et al. [39] designed the Soma Mat and Breathing Light to subtly encourage participants’ exploration of and better somatic awareness of their own bodies. Yamauchi, M. et al. [40] investigated whether environmental light affected autonomic control of heart rate, sleep-disordered breathing (SDB), and/or breathing patterning. The results show that sleeping in the light has effects like those of a stressor as it is associated with neuroexcitation, SDB, and resting breathing irregularity in healthy volunteers.

Breathing is thought to be a regulator of heart rate variability (HRV), stress perception, and chemoreflexes [41]; the central respiratory drive mechanism is capable of sensitive changes to emotions [42]. According to current studies, when people are stressed, their respiratory rate, ventilation per minute, and breathing irregularity increase [43]. The body takes longer to exhale and pauses for shorter periods of time when experiencing stressful emotions [44]. Based on breathing signals, the average accuracy of identifying human emotions can reach 80.22 percent [45]. Simultaneously, breathing has a significant impact on heart rate—the depth of breathing influences the level and variability of heart rate. Deep breathing increases heart rate and variability, whereas shallow breathing decreases heart rate levels and variability [46,47].

According to neurophysiological studies, short periods of deep breathing can help reduce the voluntary response to stressful stimuli [48]. Breathing strategies that have been specifically assessed and trained over time may be beneficial for mental health treatment [49]. Mindfulness, yoga, and specific breathing techniques can be used to relieve stress quickly and effectively in the moment [50,51]. Cardiorespiratory synchronisation during meditation appears to improve cell membrane potential homeostasis in neurons and other cells throughout the body [52]. Breathing alignment is thought to improve cardiorespiratory function and mood while increasing plasma melatonin [53]. Ban et al. [54] have also created padded devices that reveal breathing motions to adjust the user’s breathing rate for relaxation. Device-guided slow breathing (DGB) home devices that direct the breath have also been studied and employed in the treatment of chronic disorders [55].

### 1.3. Research Question

Although current studies have confirmed the strong relationship between the central respiratory system and mood, the role of breathing has been largely overlooked. These modulations, however, need targeted activity and conscious control, as well as a high level of subjective awareness in the population. Thus, how to create subliminal treatments for people via the creation of specific environments has been a focus of research in a variety of fields. Cherniack et al. [56] suggested that the sensation of breathing is determined by light and sound, while sensory stimulation and mental activity can modify breathing patterns. Masaoka and Homma [57] used unpleasant noises to demonstrate individual differences in breathing patterns under mental stress and physical pressure, confirming that negative emotions influence breathing patterns.

This study proposes that adding the breathing mechanism to a light environment can interact with users, which may make a positive influence on mood regulation. To better control the variables, this research was conducted in the evening, focusing on the artificial light environment. Mood changes are usually accompanied by changes in physiological parameters such as skin conductance and heart rate [58,59]. This study investigated the effects of light temperature and light breathing processes on self-perception (valence and arousal) and physiological indicators (ECG, pulse, and EDA), as a preliminary attempt at innovative use of light.

## 2. Material and Methods

### 2.1. Participants and Design

This experiment involved 20 participants (Mean age = 20.25, SD = 1.41; 8 males). These participants were recruited at a public university. They were asked to complete the Self-Rating Anxiety Scale (SAS) before the experiments. We selected participants whose scores were below 60 in order to prevent the possible influences brought by their own mental illness.

A 2-by-2 between-subjects experiment was designed with light breathing (presence vs. absence) and light temperature (warm vs. cold) as independent variables. Participants were firstly assigned to either cold or warm light conditions. Next, in each condition, half the participants experienced breathing while the other group did not experience light breathing. The participants in each condition can be found in Table 1.

### 2.2. Measurements

The influence of the spectral conditions on mood and cognition guidance was measured by both physiological indicators and subjective self-report scales.

#### 2.2.1. Physiological Monitors

To capture neurophysiological activity, cardiac and dermal electrical signs were employed to identify emotions, along with the SAM scale for emotion categorization. Skin conductance (SC), heart rate (HR), electrocardiogram (ECG), and respiration change in response to emotional intensity [60]. When people are emotionally stimulated, they unconsciously sweat, and their skin electricity changes [61]. EDA measurements can aid in the detection of unconscious emotions [62]. These unconscious emotions, in turn, have the potential to influence behaviour [63].

The Acknowledge 5.0.4 application software and the BiopacMP160 multichannel physiological recorder were used to capture the information from this experiment. ECG (electrocardiogram) and EDA (electrodermal conductance) measurements were taken using the PPGED and RSPEC modules, respectively. In order to limit the interference factor for the individuals, a 35 hz low-pass filter was used for ECG data capture and a 10 hz low-pass filter was used for EDA data acquisition. R-R heart rate is calculated from peak ECG monitoring through the built-in features of Acknowledge 5.0.4.

#### 2.2.2. Subjective Mood Scale

**Figure 1:**
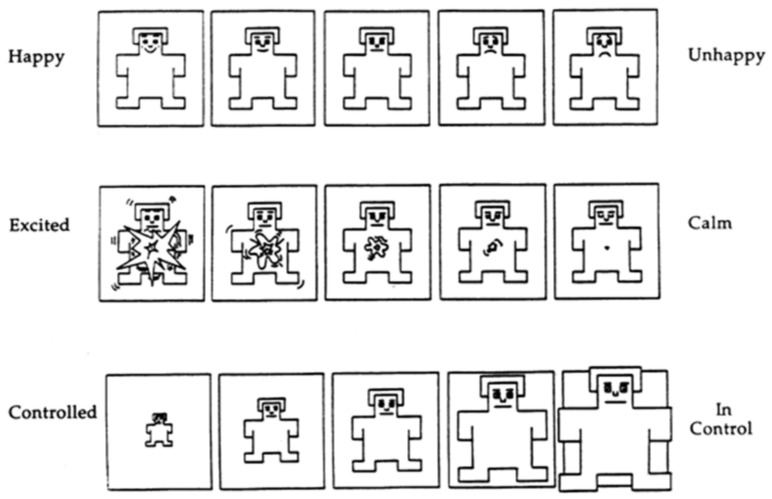
SAM Self-Assessment Manikin.

**Figure 2:**
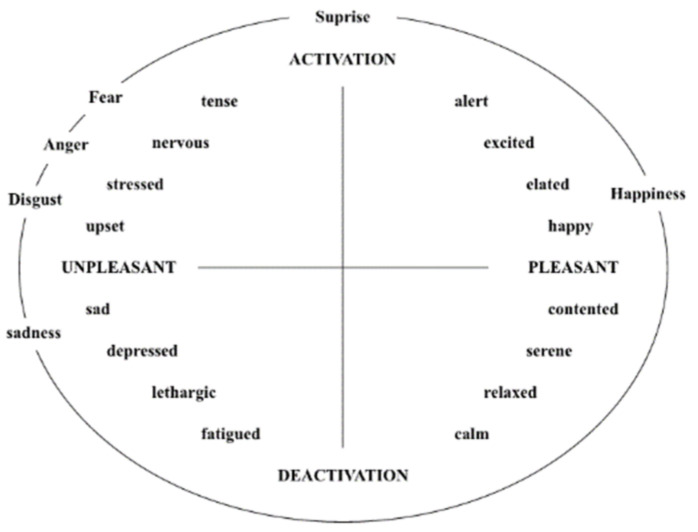
Two-Dimensional Emotion Classification.

As depicted in Figure 1, the Self-Assessment Manikin (SAM) is a nonverbal graphical assessment approach that directly evaluates pleasantness, arousal, and dominance in connection to a person’s emotional response to diverse stimuli [64]. Each dimension represents an object, and the degrees of emotional state are represented by five manikins. The assessor compares his or her own emotional state to that of the manikin, selecting the suitable model or a state between the two models and scoring it on a scale of 1–9. The first row from left to right indicates a drop in pleasure (being less joyful), the second row from left to right indicates a decrease in arousal (getting less enthusiastic), and the third row from left to right indicates an increase in dominance (becoming assertive). Pleasure and arousal indicated by SAM have been shown to be strongly correlated with physiological and behavioural responses. Lang [65] examined how valence and arousal may be used to categorize emotions in a two-dimensional space. Valence, according to his hypothesis, spans from unpleasant (negative) to pleasant (positive), while arousal, which reflects how intensely persons feel, ranges from passive (low) to active (high). Because different emotions can be plotted in these two dimensions, as shown in Figure 2, the study used the first two SAM measures as emotion scales to record individuals’ emotional states.

### 2.3. Emotion Arousal Stimuli Creation

Prior research has demonstrated that a film or story is most successful in eliciting both good and negative emotional states [66]. Compared with stories, films have relatively high ecological validity; in addition, emotions are often evoked by external dynamic visual and auditory stimuli [67]. Three film clips were chosen for pre-testing to ensure that specific negative emotions could be induced. The selection of final video clips in this experiment was based on the stimulating effect on the individuals.

To assess the film’s effectiveness, we conducted a pre-test to compare participants’ heart rate, skin electrical value, pleasure, and excitement before and after viewing. Through a paired sample t-test, the results revealed that the 8 min video skit was the most effective for emotion induction, with participants’ heart rate significantly increased (*t* = −2.25, *p* < 0.05); the skin electrical value also significantly increased (*t* = −5.23, *p* < 0.001). Subjective scores also revealed significant differences in terms of subjective pleasure (*t* = 2.60, *p* = 0.017) and arousal (t = −8.23, *p* = 0.000). These results confirmed the effectiveness of created stimuli for triggering negative emotions.

### 2.4. Experimental Setting

**Figure 3:**
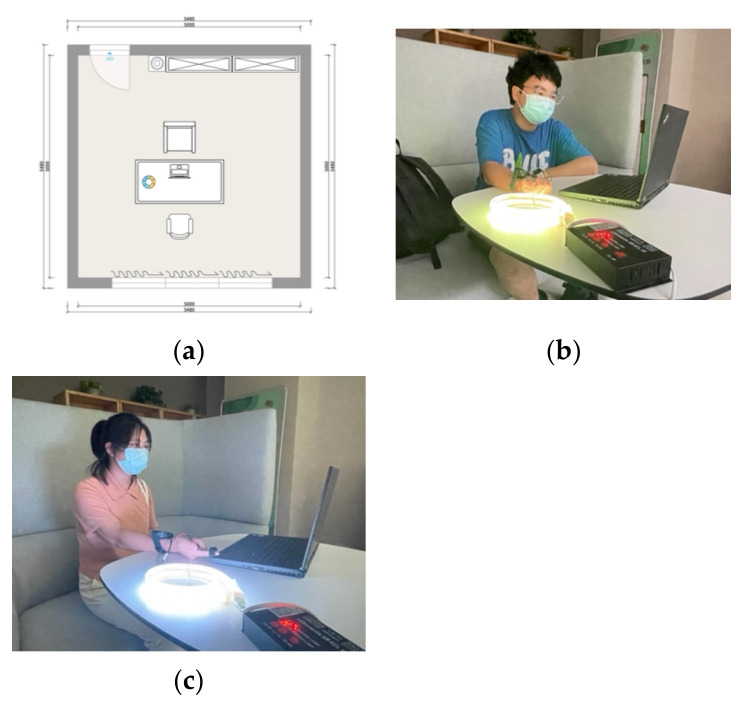
The setting and LED light installation. (**a**) The experiment was carried out in an enclosed space of 25 m^2^, with only a desk and a light at a distance of approximately 800 mm from the participants’ eyes. (**b**) Participant immersed in the warm light environment. (**c**) Participant immersed in the cold light environment.

The trials were carried out in a 5 m * 5 m enclosed room, with the overall spatial distribution depicted in Figure 3. The experiments were conducted in the dark with light levels less than 10 lx. Experimental LED light was the only source of light in the room. Throughout the experiment, the external temperature was kept constant at 18–20 degrees. The experimenter and position were fixed during the experiment.

**Figure 4:**
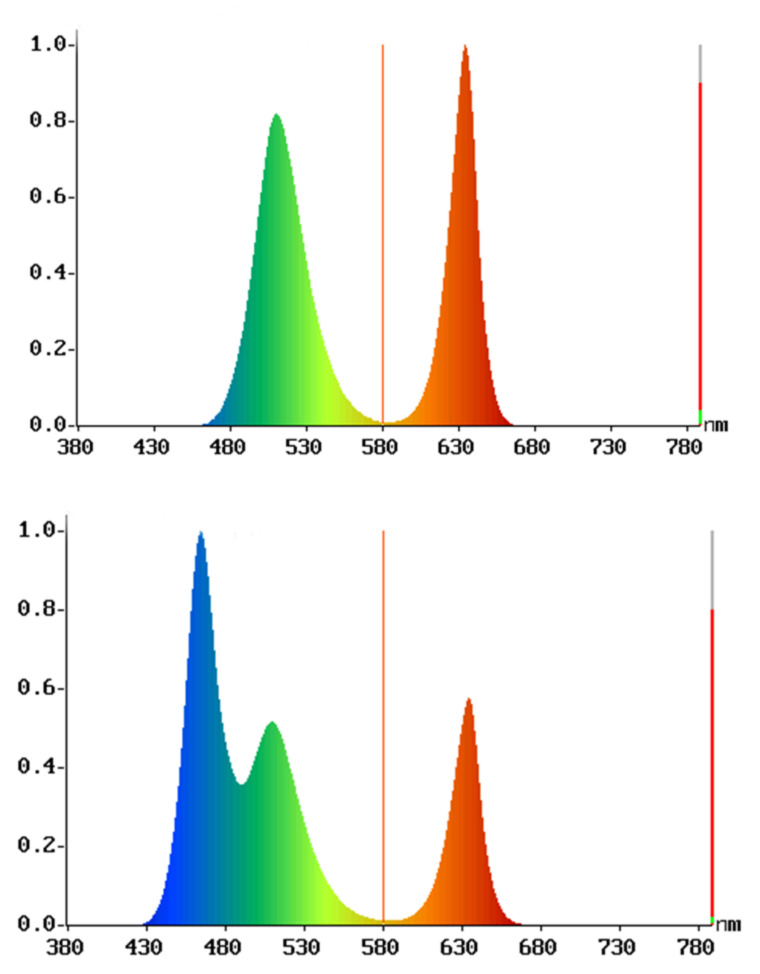
Spectrograms of warm and cold light environments.

The OHSP-350 spectrometer was used to measure the light intensity, spectral power distribution (SPD), and colour rendering index (CRI) of the participant’s eye. The brightness of the light fixture was approximate 20 lx, and it was placed on the table at a distance of 1 m from the participants. The warm light had a colour temperature of 5000 k and a wavelength of 560 nm. The cold light had a colour temperature of 10,000 k and a wavelength of 480 nm. The spectral diagrams of the two are shown in Figure 4.

Breathing is an essential sign for monitoring sleep and individual health. Thus, [68] used sensors to assess the breathing rate of family members ranging in age from 9 months to 69 years during nocturnal sleep and reported people in their 30s taking 12–15 breaths per minute at night. For the best results, the lowest respiratory rate value was chosen for higher guided emotional calm and the experimental breathing light flashes were set to 5 s cycles.

### 2.5. Procedure

Three days prior to the main experiment, all participants were instructed to get adequate sleep and rest. On the day of the experiment, we sent them a reminder to arrive at the lab on time. Before entering the lab, the participants were informed about the procedure and precautions, and they were asked to complete an informed consent form. Next, they were instructed to wear the ECG and EDA devices and entered the lab for the main session. Participants were exposed to light immersion for approximately 20 min at the same time of night (6.00 p.m., 6.50 p.m., 7.40 p.m., and 8.20 p.m.). The order of the individuals was randomized but the experimental sequence and geographical surroundings remained the same.

**Figure 5:**
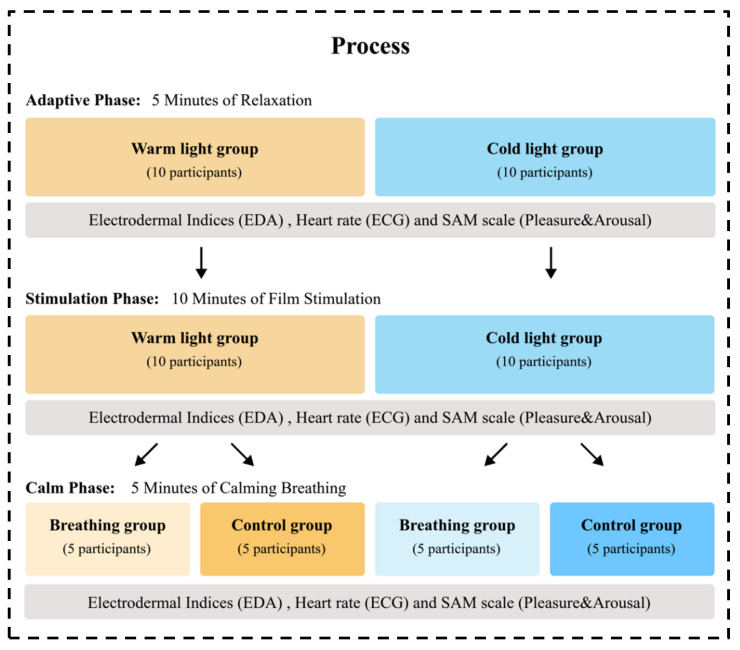
Experimental process.

The procedure of the main experiment is visualized in Figure 5. In the first stage, when participants entered the test room, the lighting environment was fully set up and the experiment began. Subjects were given 5 min to adjust to the atmosphere. Next, they were asked to fill in the SAM scale. After participants confirmed that they have been familiar with the experimental environment, the second stage started.

Participants were asked to watch the created film that was intended to evoke anxiety emotions. This film lasted approximately 10 min. At the end of the film, participants were asked to fill in another SAM scale to indicate their emotions. At this stage, participants’ emotions were supposed to be aroused by the film. In the third stage, the experimental intervention of a breathing light was involved. The light was either set to a breathing mode or no-breathing mode. This breathing or no-breathing lighting condition lasted for 5 min, which aimed to calm the participants. After 5 min experience of the lighting condition, participants were asked to fill in the SAM scale. The experiment lasted approximately 20 min in total. Throughout the three stages of the experiment, participants wore the physiological monitors.

### 2.6. Data Analysis

SPSS Statistics 26.0 was used to conduct statistical analysis on the experiments. In order to test the effect of the breathing light, we used a generalised linear mixed model (GLMM) with significance analysis for the respiratory and control groups. Moreover, the two-way test for light colour (warm vs. cold) and breathing (breathing vs. static) was used to investigate whether breathing lights of different light colours had equivalent effects.

## 3. Results

The generalised linear mixed model (GLMM) analysis was conducted to analyse the data in the stimulation phase and the calm phase. The physiological data revealed significant differences between the breathing group and the control group but not between the different lighting conditions.

**Figure 6:**
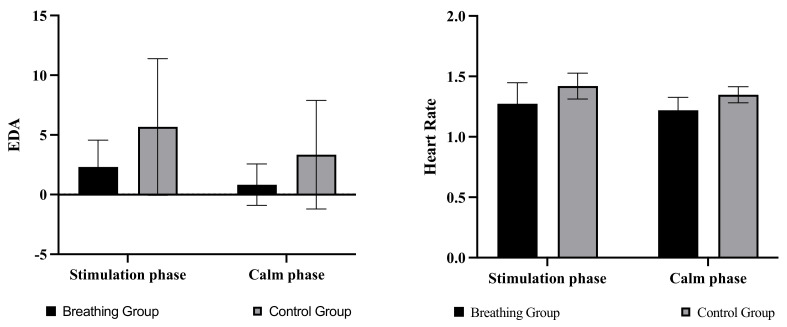
EDA values and Heart Rate for the respiratory and control groups during the stimulation and pacification phases.

Results revealed that in comparison with the control group, the breathing group showed a significant decrease in both EDA values and heart rate. The mean EDA values were 2.85 micro-ohm smaller in the respiratory group compared with the control group (*p* = 0.007. Mean = 4.29 vs. mean = 1.44). In addition, the heart rate values were also 0.13 break/s smaller than the control group (*p* = 0.002. Mean= 1.34 vs. mean = 1.22). These physiological data suggest that the lighting’s breathing can help lower heart rate and emotional stimulation [69] (see Figure 6 for results). These results indicated that the breathing effect was effective in guiding participants’ physiological regulation in both cold and warm lighting environments.

In terms of subjective ratings, no significant differences were detected between the two groups (*p* > 0.100), despite some participants reporting that the flashing of the breathing light did make them feel relaxed and comfortable. This could also indicate that physiological data is more sensitive than subjective ratings.

To further investigate whether different colours of respiratory light have the same effect, we conducted a two-way test of the different light temperatures (warm light and cold light) for each of the two groups (breathing group and control group). The results of the data are shown in Table 2. The breathing group’s results revealed that the light colour temperature had little effect on the breathing effect and showed significant differences only in subjective arousal scores. Participants exposed to warm light rated their arousal 1.38 points higher on average than those exposed to cold light (*p* = 0.043). This indicated that participants perceived the breathing effect of the cold light to be more effective in calming alertness. No other effects were detected.

## 4. Discussion

This study investigated the calming effect of breathing lights on extreme negative emotions. The experiment began with negative mood arousal in the population, and then used breathing lights to assess physiological indicators as well as subjective mood changes in the population, yielding the key results listed below.

To begin, the findings of this study show that breathing lights can significantly reduce electrodermal values as well as heart rate in post-stimulus population. Immersion in a breathing light environment reduced the participants’ EDA value and their heart rate. This could be related to the population’s subconscious breathing regulation. According to some reports, the autonomic nervous system (ANS) is regulated by breathing; therefore, in sympathetic dominant situations such as feeling stress and anxiety, slow deep breathing can shift sympathetic dominance to parasympathetic dominance, increasing cell membrane potential homeostasis and lowering excitability of the heart, the amygdala, and other pacemakers [70,71]. Noble et al. [72] also revealed the benefits of counteracting autonomic sympathetic bias and lowering responsiveness to stresses. In line with this, results of this study demonstrate that breathing lights were helpful in directing and lowering participants’ heart rates under untrained situations, as well as relaxing intense emotions in a short time.

We also discovered that the colour temperature of the light had no effect on this effect. In terms of both EDA and ECG signals, there were no significant differences in the respiratory effect between the cold and warm colour temperature groups. This appears to contradict previous research findings. The effect of CCT on perception was objectively studied by Chao et al. [5], and it may be related to the high sensitivity of ipRGCs to short wavelength light. Notwithstanding, some researchers demonstrated that cognitive and emotional behavioural outcomes do not differ significantly among warm white light, cold white light, and full-spectrum environments [73].

The results did not reveal significant differences on subjective perception. This indicates that the effect of light breathing happens unconsciously. This could be due to the slow flashing frequency of the lights, which causes participants to slow their breathing [74]. Breathing lights have been shown to reduce heart rate and EDA values. Previously, the EDA index was found to have a positive relationship with arousal [75]. However, in the current study, there was no difference in subjective scores between the breathing and control groups. This result indicates that the participants did not, or to a lesser extent, perceive the value of the effects of light.

## 5. Conclusions

Breathing light in 5 s cycles can significantly reduce EDA values and heart rate in people experiencing extreme emotions, thereby reducing tension. Colour temperature had little to no effect on the effectiveness of breathing light interventions. In comparison with the colour temperature of the light, the effect of slow breathing will be more intuitive for the crowd to experience. Furthermore, this study showed that breathing lights can assist with emotional relaxing; the usage of frequency should be examined further. Meditation is one of the most essential methods of controlling one’s breathing, and its high degree of cardiorespiratory synchronization (4–5 heartbeats per breath) is considered to support its impact on emotional states [76,77]. This also implies that variances in heartbeat breathing between persons must be further taken into account in order to arrive at tailored solutions, and, of course, with due regard for the user’s privacy choices [78]. In general, the impacts of breathing lights on human therapies require further particular and in-depth research.

## Figures and Tables

**Table 1:** Distribution of participants.

Colour Temperature	Breathing Group	No-Breathing Group
Cold light	5	5
Warm light	5	5

**Table 2:** Physiological values and subjective scores of the respiratory and control groups in different colour temperature environments during the stimulation phase and the calming phase.

Breathing Condition	Light Condition	Statistics	EDA	Heart Rate	Pleasure	Arousal
Breathing group	Warm light	Mean	2.73/0.45	1.28/1.21	4.60/5.20	6.20/4.40
		SD	1.98/2.03	0.18/0.11	2.61/1.10	1.10/0.89
	Cold light	Mean	1.91/1.22	1.22/1.19	4.00/5.80	5.80/2.80
		SD	2.63/1.49	0.21/0.14	1.00/2.17	1.64/1.10
Control group	Warm light	Mean	9.44/6.13	1.47/1.34	4.20/4.80	5.40/2.60
		SD	5.63/4.95	0.11/0.08	1.79/0.45	1.67/0.89
	Cold light	Mean	1.89/0.55	1.30/1.31	4.60/5.40	6.20/4.20
		SD	2.53/1.64	0.08/0.10	4.60/1.14	1.10/1.30

## Data Availability

Some or all of the resulting data are available upon request.
